# PVA/κ-carrageenan/Au/camptothecin/pegylated-polyurethane/paclitaxel nanofibers against lung cancer treatment

**DOI:** 10.1039/d2ra02150a

**Published:** 2022-06-01

**Authors:** Mohammad Irani, Sina Mohammadrezaei Nodeh

**Affiliations:** Faculty of Pharmacy, Alborz University of Medical Sciences Karaj Iran irani_mo@ut.ac.ir

## Abstract

Gold nanoparticles, paclitaxel (PTX), and camptothecin (CMPT) were loaded into the PVA/κ-carrageenan/pegylated-PU composite and core–shell nanofibers prepared by two-nozzle and coaxial electrospinning methods. The capability of composite and core–shell nanofibers was investigated for the targeted delivery of anticancer drugs in lung cancer treatment. *In vitro* and *in vivo* release of PTX and CMPT were investigated to find the release mechanism from nanofibers compared to direct administration of pristine PTX and CMPT. The mean fiber diameter for composite and core–shell nanofibers with shell feeding rates of 0.3, 0.5, and 0.7 mL h^−1^ was about 225, 330, 520, and 640 nm, respectively. *In vivo* release studies indicated that the blood concentration of CMPT and PTX for rats fed with core–shell nanofibers reached the highest values of 26.8 ± 0.04 μg mL^−1^, and 26.5 ± 0.05 μg mL^−1^ in 36 h, and 24 h and reduced slowly within 84 h, and 48 h, respectively. The maximum cytotoxicity was 75% in the presence PVA/κ-carrageenan/CMPT/Au/pegylated-PU/PTX core–shell nanofibers. *In vivo* antitumor activity results confirmed the synergic effect of Au, CMPT and PTX anticancer drugs on the reduction of tumor volume without change in mouse weight by the PVA/κ-carrageenan/CMPT/Au/pegylated PU/PTX core–shell nanofibers. The obtained results indicated that the simultaneous loading of CMPT and PTX anticancer drugs and Au nanoparticles is more beneficial for lung cancer treatment.

## Abstract 2

Core–shell nanofibers and *in vivo* release from core–shell nanofibers against lung cancer.

## Graphical abstract

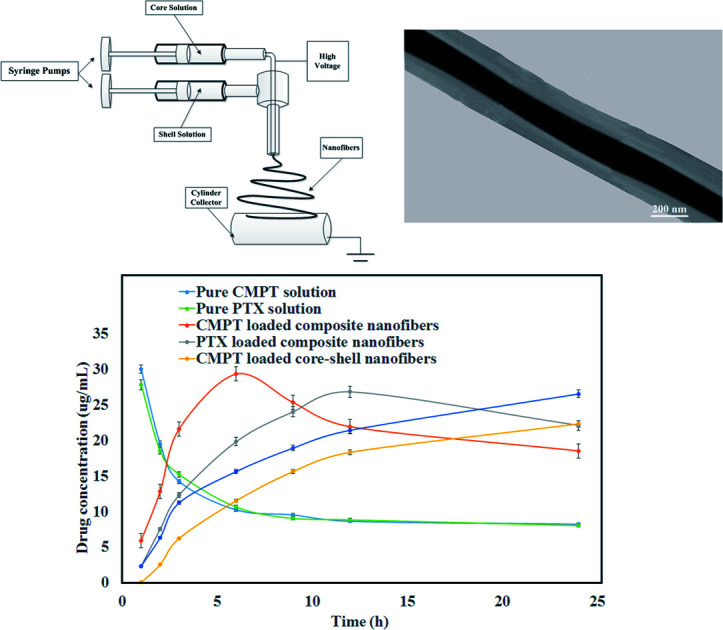

## 1. Introduction

The combination of paclitaxel (PTX) and camptothecin (CMPT) could provide several advantages over monotherapy. In previous studies, better treatment of various cancers, including lung, breast, and melanoma, is demonstrated by the co-delivery of PTX and CMPT compared to the monotherapy of PTX or CMPT1-4. For instance, the concentrations of PTX and CMPT could be controlled by their loading into the liposome.^1^ Borrelli *et al.*^2^ synthesized the squalene conjugates with PTX, CMPT, podophyllotoxin, and epothilone against A549 lung cancer cells. In other study, the combined effect of PTX and CMPT was investigated against melanoma,^3^ breast, pancreatic,^4^ and medullary thyroid carcinomas.^5^ Chen *et al.*^6^ attached PTX and CMPT anticancer drugs on the 2-nitroimidazole against hypoxic tumors. In another study, CMPT and PTX were conjugated with two cyclic cell-penetrating peptides to inhibit MCF-7 breast cancer cells.^7^ Hariri *et al.*^8^ investigated the targeted delivery of PTX and CMPT from a cross-linked sponge against lung cancer. The use of targeted drug delivery systems such as liposomes,^9,10^ microspheres,^11,12^ nanoparticles,^13,14^ and nanofibers^15–18^ could provide the sustained delivery of anticancer drugs.

κ-Carrageenan, an anionic sulfonated polysaccharide, is extracted from red algae and used for biomedical applications such as food, tissue engineering and drug delivery systems.^19–23^ Carrageenan could also interact with inorganic materials such as graphene oxide (GO), metal–organic frameworks (MOFs) and proteins. κ-carrageenan, and κ-carrageenan-based composites have been used in various drug delivery systems. For instance, Sun *et al.*^20^ interacted κ-carrageenan with zein nanoparticles. Javanbakht *et al.*^21^ synthesized κ-carrageenan/MOFs composite hydrogel for oral delivery. Vinothini *et al.*^22^ used κ-carrageenan-grafted graphene oxide for drug delivery of DOX. Tort & Acartürk^23^ investigated the capability of electrospun κ-carrageenan nanofibers for oral mucositis treatment. The use of nanofibers prepared by electrospinning technique is an effective strategy for loading the high content of drugs due to their high porosity, high surface area, small size, good flexibility, and interconnecting channels.^24^ However, there are still limitations for electrospun nanofibers, due to their rapid degradation over time. The various nanofibers, including composite, multi-layered,^25^ and core–shell nanofibers,^17,18^ could provide the prolonged-release profiles. In recent studies, the hydrophobic polymers such as poly(ε-caprolactone),^26^ polyurethane (PU),^27^ poly(lactic acid) (PLA),^28^ and poly(lactic-*co*-glycolic acid) (PLGA)^29^ were used to increase the stability of polysaccharide nanofibers in aquatic systems.

Most PUs are non-degradable and have little use in biomedical applications. On the other hand, the natural/synthetic biodegradable polymers are stiff with low flexibility or soft with poor mechanical properties. To overcome these challenges, the biodegradable PUs, composed of a hard segment, soft segment, and chain extender, have been introduced as one of the most popular polymers due to their low cost, high biocompatibility, and excellent efficiency for drug delivery systems.^30^ The degradation rate of PUs is mainly due to the cleavage of hydrolytic bonds present in their soft segments. The composition of the soft segments could control the degradation rate of the PUs. The degradation rate of PUs with hydrophilic soft segments such as poly(ethylene glycol) is faster than PUs with hydrophobic soft segments such as polycaprolactone diol.^31^ Loading of anticancer drugs into the PUs, and its derivates could increase the availability of the anticancer agents at the tumor sites, suppress the cancer cells that did not kill by the initial dose of drug^31,32^ and decrease the adverse side effects. For instance, Gajbhiye *et al.*^33^ reviewed the performance of biodegradable PU nano-constructs for the targeted delivery of anticancer drugs. Yin *et al.*^34^ indicated that PTX molecules were released slowly from liposome-encapsulated PU scaffolds compared to PTX release from PU. Liu *et al.*^35^ investigated the potential PU functionalized with amine, and carboxyl group micelles for incorporating of doxorubicin hydrochloride (DOX) and PTX. In previous studies, the capability of drug-loaded PU nanofibers for various cancers treatment was studied by our group.^17,18,36,37^ Recently, inorganic materials such as metal oxides, zeolites, MOFs and noble metals were loaded into the nanofibrous matrix to increase the delivery of anticancer drugs on the surface of the cancerous tissues. Gold-based nanomaterials with various shapes, including nanoparticles,^38–40^ nanocages,^41,42^ and nanorods^43–45^ are incorporated into the different forms of drug delivery systems for targeted delivery and imaging in cancer therapy. Gold nanoparticles (Au NPs), due to their high stability, and easier synthesis were used to improve the attachment of cancerous cells and increase the therapeutic efficacy.^46,47^

In the present study, PTX and CMPT were loaded into the κ-carrageenan/Au/pegylated-PU composite nanofibers prepared by two-nozzle and coaxial electrospinning methods for targeted delivery of anticancer drugs against lung cancer treatment. It was hypothesized that the co-delivery of PTX and CMPT from nanofibers and the use of gold nanoparticles in the nanofibers structure could enhance the targeted delivery of anticancer drugs on cancerous tissues.

## 2. Experimental

### 2.1 Materials

Poly(ethylene glycol) (*M*_n_ = 2000 Da, PEG) supplied from Sigma-Aldrich (USA); hexamethylene diisocyanate (HDI) and 1,4-butanediol (BDO) purchased from Merck Company (Germany) were used to synthesize the pegylated polyurethane. Polyvinyl alcohol (99% hydrolyzed, *M*_n_ = 72 kDa) and κ-carrageenan were used to prepare PVA/κ-carrageenan nanofibers. Paclitaxel and camptothecin supplied from Sigma-Aldrich (USA) were utilized as anticancer drugs. HAuCl_4_, and sodium citrate provided from Sigma-Aldrich (USA) were applied to synthesize the gold nanoparticles.

### 2.2 Preparation of core–shell solutions

The shell solution was the PTX-loaded pegylated polyurethane (PU). The pegylated polyurethane was synthesized by reacting PEG, HDI, and BDO as described previously by Yao *et al.*^48^ 10 wt% pegylated PU electrospun solution was prepared by dissolving in *N*,*N*-dimethylacetamide (DMF, Merck). To load PTX molecules into the pegylated PU, 1 and 2 mg mL^−1^ PTX were added into the pegylated-PU solution under sonication for 30 min.

The core solution was the CMPT/Au-loaded PVA/κ-carrageenan solution. The citrate reduction of HAuCl_4_ was used to synthesize Au nanoparticles.^37^ 2 wt% κ-carrageenan (dissolved in distilled water) and 10 wt% PVA (dissolved in distilled water) were mixed under stirring for 6 h (3 : 7 v/v). To load CMPT and Au nanoparticles into the PVA/κ-carrageenan solution, 2 wt% Au suspension (with respect to the PVA/κ-carrageenan solution v/v) and 1, 2 mg mL^−1^ CMPT were added into the PVA/κ-carrageenan solution under sonication for 30 min.

### 2.3 Preparation of composite nanofibers and core–shell nanofibers

To prepare PVA/κ-carrageenan/CMPT/Au/pegylated PU/PTX composite nanofibers, the prepared pegylated PU/PTX and PVA/κ-carrageenan/CMPT/Au solutions were separately transferred into the two syringes (needle tip: 19 gauge) and then placed at both counter sides of the cylindrical collector which was placed an aluminum foil on the collector. The applied high voltage was varied between 15 and 25 kV. Tip–collector distance was constant at 12 cm. The feeding rate was between 0.3–1 mL h^−1^ for both solutions to produce the PVA/κ-carrageenan/CMPT/Au/pegylated PU/PTX composite nanofibers under an electrospinning time of 4 h.

To prepare the PVA/κ-carrageenan/CMPT/Au/pegylated PU/PTX core–shell nanofibers, the prepared core and shell solutions were transferred into the two syringes (tip needle: 27 and 14 gauges). The voltage, tip–collector distance, and core solution feeding rate were constant at 20 kV, 12 cm, and 0.3 mL h^−1^. The shell solution feeding rate varied between 0.3 and 0.7 mL h^−1^.

### 2.4 Instrumentation

Fourier transform infrared (FTIR) analysis was implemented ranging from 500–4000 cm^−1^ with a resolution of 2 cm^−1^ on the Fourier-transform infrared spectroscopy (Brucker, Tensor 27, Germany). X-ray diffraction (XRD) analysis ranged from 5° to 80° using an X-ray diffractometer (Intel, EQUINOX3000, France). Morphology of nanoparticles and nanofibers was carried out using a scanning electron microscope (SEM, AIS2100, SERON Technology, Republic of Korea) and a transmission electron microscope (TEM, H9500, Hitachi, Japan).

### 2.5 Drug loading efficiency

To evaluate the drug loading measurements in the nanofibers, drug entrapment efficiency (DEE%) and drug loading efficiency (DLE%) were calculated as follows:1
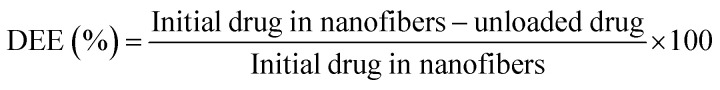
2
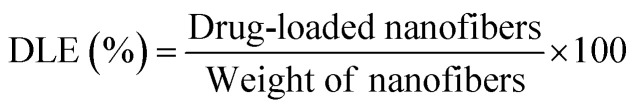


### 2.6 *In vitro* drug release and pharmacokinetic studies

*In vitro* release studies were implemented by immersion of 100 mg drug-loaded nanofibers in 500 mL of phosphate-buffered saline (PBS) solutions with a rotation rate of 50 rpm at the temperature of 37 °C, and pH values of 5.5 and 7.4. At preplanned times, 2 mL of aliquot was withdrawn, and 2 mL of fresh PBS was added. The final concentrations of PTX and CMPT were determined using High-performance liquid chromatography (HPLC, Agilent 1260, USA) and UV/Vis spectrophotometer (JAS.CO V-530, Japan) at 230 nm, and 360 nm, respectively. HPLC conditions are: a reversed-phase HPLC method, column: C-18 column, mobile phase: acetonitrile, methanol and water (50 : 10 : 40, v/v), temperature: 25 °C and flow rate of 1.0 mL min^−1^. Experiments were repeated three times, and the results were reported as mean ± S.D. The experimental data was statistically analyzed using an analysis of variance (ANOVA) as well as a *t*-test (*P* < 0.05).

The release data were fitted by pharmacokinetic models, including zero order (*Q*(*t*) = *K*_0_*t*), Higuchi (*Q*(*t*) = *K*_H_*t*^0.5^), and Korsmeyer–Peppas (*Q*(*t*) = *At*^*n*^) equations.

### 2.7 *In vivo* release studies

For investigating the *in vivo* release of PTX and CMPT from nanofibers, first, the drug-loaded nanofibers were implemented into the 15 old mice with an average weight of 25 g (Tehran University) that were maintained under a maximum isolation environment for 6 weeks based on Institutional Animals Ethics Committee (94/PO/ReBi/S/1999/CPCSEA). All animal procedures were performed in accordance with the Guidelines for Care and Use of Laboratory Animals of Tehran University and approved by the Animal Ethics Committee of National Institute for Medical Research Development (NIMAD), Tehran, Iran. Then, nanofibers-implemented 15 old mice were divided into five groups of three (pure PVA/κ-carrageenan/pegylated PU nanofibers, PVA/κ-carrageenan/Au/pegylated PU nanofibers, PVA/κ-carrageenan/CMPT/Au/pegylated PU core–shell nanofibers, PVA/κ-carrageenan/CMPT/Au/pegylated PU/PTX core–shell nanofibers and PVA/κ-carrageenan/CMPT/Au/pegylated PU/PTX composite nanofibers). The mice dose of PTX and CMPT was 5 mg kg^−1^. The nanofibrous scaffolds were implanted into the lower right flank of each mouse. At predetermined intervals (1, 2, 3, 6, 12, 24, 36, 48, 60, 72, 84 and 96 h), 0.5 mL of blood samples were withdrawn and were stored in a centrifuge tube. The samples were centrifuged at 5000 rpm for 5 min to separate the plasma that was frozen at −20 °C until HPLC and UV assays to determine the concentrations of PTX and CMPT, respectively.

### 2.8 Cell viability

To investigate the biocompatibility, and cytotoxicity of nanofibers, the pure drugs and drugs-loaded nanofibers were cut into flakes and placed in each well of L929 murine fibroblast and A549 lung cancer cell lines. Cells were purchased from the Institute Pasteur of Iran, and cultured in RPMI-1640 media under 5% CO_2_ and 37 °C for 24, 48 and 72 h. 5 × 10^4^ cells per well were transferred in a 48-well plate and treated with 1 and 2 mg mL^−1^ concentrations of pure drugs and their equivalent doses of drugs-loaded nanofibers. The MTT assay was implemented by using a microplate reader (Multiskan MK3, Thermo Electron Corporation, USA) at the wavelength of 570 nm.^27^ Briefly, the MTT assay was carried out as follows: 20 μL of MTT solution with a concentration of 5 mg mL^−1^ in PBS (pH 7.4) was added to each well, and then, were incubated for a further 4 h. Then, the solution was aspirated cautiously from each well. After treating the cells with Sorenson buffer, the optical density of each well was readied at 570 nm.

### 2.9 Antitumor efficiency *in vivo*

The tumor-bearing mice were divided into 5 groups of three. 2 × 10^6^ A549 lung cancer cells were subcutaneously injected into the left hind flank of each anesthetized mice after isolation for 6 weeks. When the tumor volume reached 100 mm^3^, the mice were treated with nanofibrous samples, and experiments were continued for 20 days. The relative tumor volume with respect to the initial volume of tumor (*V*_0_ = 100 mm^3^) before treatment was calculated as *V*/*V*_0_. Briefly, animals were anesthetized by intra-peritoneal injection of pentobarbital at 20 mg kg^−1^, and a small incision was made on the skin to expose the tumor. A small incision was made into the tumor, and synthesized fibers were inserted into the tumor and then the wound was closed using subcutaneous suturing. The tumor volume is calculated as length × width × (length + width)/2.

## 3. Results and discussion

### 3.1 Characterization of nanofibers

**Fig. 1:**
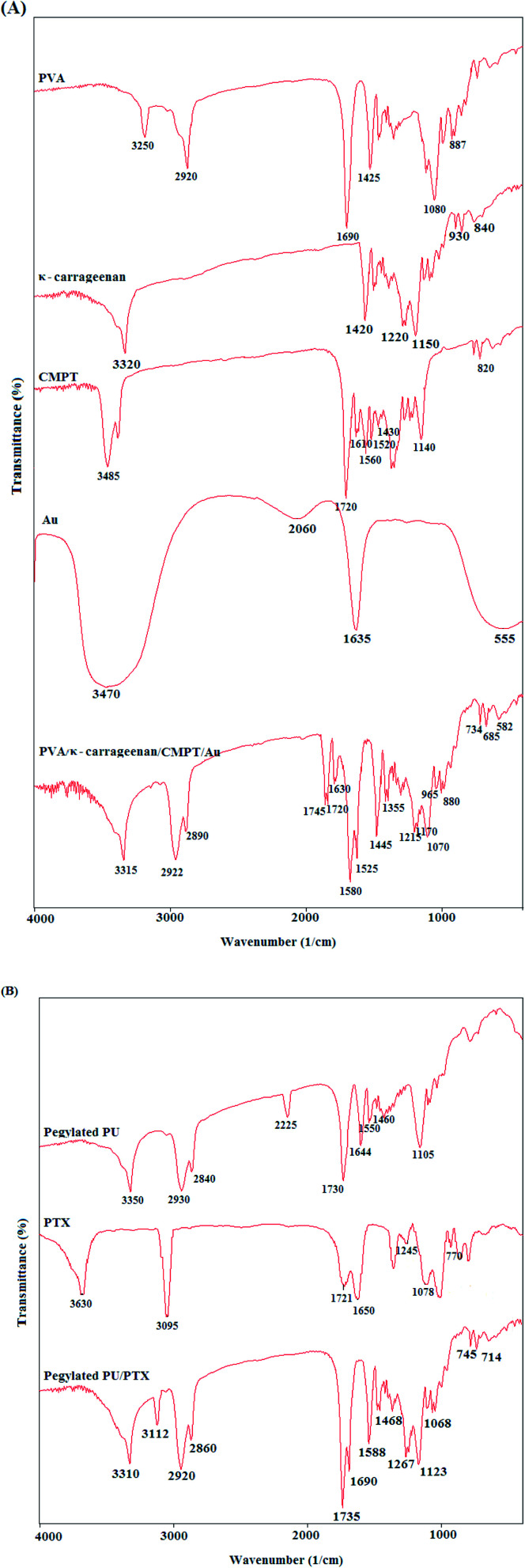
FTIR spectra of (A) PVA, κ-carrageenan, CMPT, Au, PVA/κ-carrageenan/CMPT/Au, and (B) pegylated PU, PTX, and pegylated PU/PTX.

FTIR spectra of PVA, κ-carrageenan, Au, CMPT, PVA/κ-carrageenan/CMPT/Au, pegylated-PU, PTX, and pegylated-PU/PTX are illustrated in Fig. 1. For pure PVA, the detected peaks at 3250 cm^−1^, 2920 cm^−1^, 1690 cm^−1^, 1425 cm^−1^, 1080 cm^−1^ and 887 cm^−1^ were assigned to the –OH stretching, CH_2_ asymmetric stretching, C=O stretching, CH_2_ bending, C–O stretching, and C–C stretching, respectively. For pure κ-carrageenan, the observed peaks at around 3320 cm^−1^, 1420 cm^−1^, 1220 cm^−1^, 1150 cm^−1^, 930 cm^−1^, and 840 cm^−1^ were due to the –OH stretching, C–O–H bending, O=S=O stretching, C–O–C absorption, and O–SO_3_ stretching bands, respectively. The new peaks between 500–700 cm^−1^ after loading Au nanoparticles into the PVA/κ-carrageenan confirmed the presence of Au nanoparticles in the nanofibrous matrix. The shift of carbonyl stretching vibrations correspond to the ketone groups from 1610 cm^−1^ to 1660 cm^−1^, and the lactone groups from 1720 cm^−1^ to 1745 cm^−1^ demonstrated the interaction of CMPT molecules with nanofibrous matrix. For pegylated PU, the appeared peaks at 3350 cm^−1^, 2930 cm^−1^, 2840 cm^−1^, 2225 cm^−1^, 1730 cm^−1^, 1644 cm^−1^, 1550 cm^−1^, 1460 cm^−1^, and 1105 cm^−1^ were related to the N–H stretching, asymmetric CH_2_ stretching, symmetric CH_2_ stretching, –N=C–O absorption, C=O stretching, urea absorption, N–H bending, CH_2_ bending, and C–O–C absorption, respectively. The detected peaks, in the FTIR spectrum of pure paclitaxel, at 3630 cm^−1^, 3095 cm^−1^, 1721 cm^−1^, 1650 cm^−1^, 1245 cm^−1^ and 1078 cm^−1^ were due to the presence of O–H, CH_2_, C=O, C–C, C–N and C–O groups. The prominent peaks of pegylated PU and paclitaxel have overlapped.

**Fig. 2:**
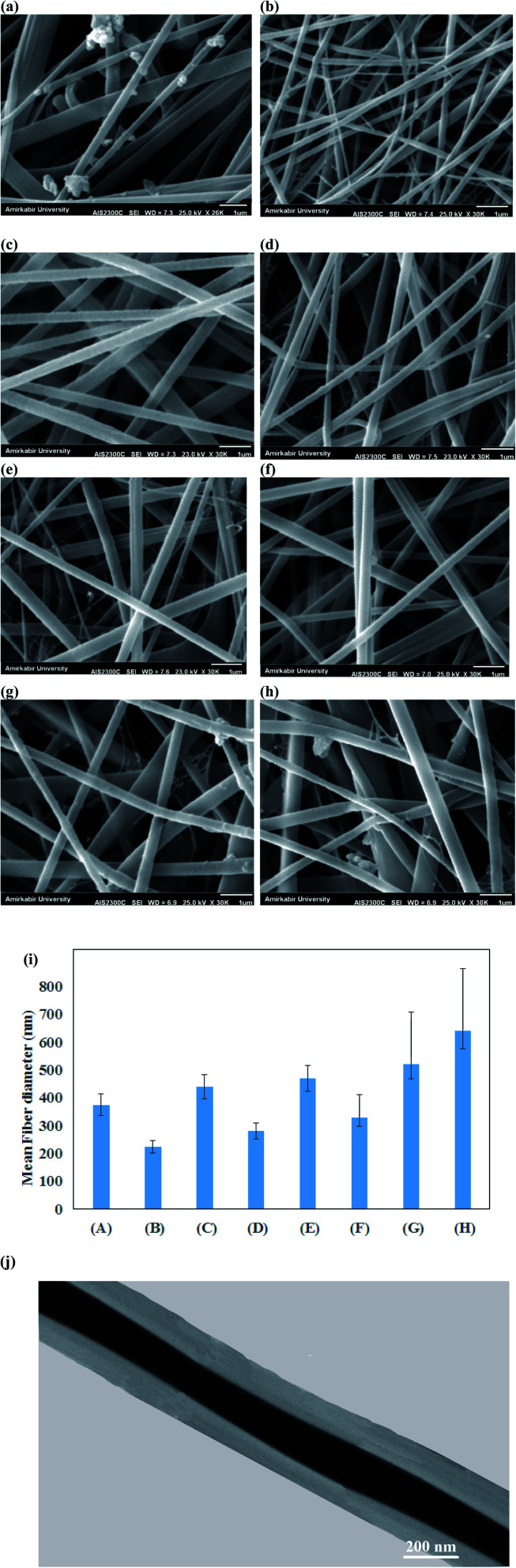
SEM images of PVA/κ-carrageenan/CMPT/Au/pegylated PU/PTX composite nanofibers under feeding rate of 0.3 mL h^−1^ and applied voltages of (a)15 kV, (b) 20 kV, (c) 25 kV and different feeding rates of (d) 0.5 mL h^−1^, (e) 1 mL h^−1^, and core–shell nanofibers with core feeding rate of 0.3 mL h^−1^ and shell feeding rates of (f) 0.3, (g) 0.5 and (h) 0.7 mL h^−1^, (i) mean fiber diameter of synthesized nanofibers and (j) TEM image of core–shell nanofibers.

SEM images of PVA/κ-carrageenan/CMPT/Au/pegylated PU/PTX composite nanofibers under different applied voltages (15, 20 and 25 kV) and feeding rates (0.3, 0.5, and 1 mL h^−1^) are presented in Fig. 2. By increasing the applied voltage up to 20 kV, the more homogeneous fibers were prepared on the collector (mean = 225 nm, Fig. 2b). The higher applied voltage (25 kV) led to instability of jet solution and enhancement of fiber diameter (mean = 440 nm, Fig. 2c). However, an increase in the feeding rate from 0.3 to 1 mL h^−1^ led to increase the fiber diameter from 225 nm to 470 nm. Therefore, an applied voltage of 20 kV, tip–collector distance of 12 cm and feeding rate of 0.3 mL h^−1^ were selected to fabricate composite nanofibers for further experiments. SEM images from PVA/κ-carrageenan/CMPT/Au/pegylated PU/PTX core–shell nanofibers with different shell feeding rates (0.3, 0.5 and 0.7 mL h^−1^), core feeding rate of 0.3 mL h^−1^, the voltage of 20 kV, and the tip–collector distance of 12 cm are presented in Fig. 2f–h. As expected, an increase in shell feeding rate from 0.3 mL h^−1^ to 0.5 mL h^−1^, and 0.7 mL h^−1^ led to increased fiber diameter from 330 nm to 520 and 640 nm, respectively. The comparison of the PVA/κ-carrageenan/CMPT/Au/pegylated PU/PTX composite and core–shell nanofibers indicated that the composite nanofibers had a sharp distribution of fibers compared with broader distribution of core–shell nanofibers at studied condition. However, the non-alignment of fibers was detected for both composite and core–shell nanofibers, and thus have a randomly distributed nanofibrous structure for synthesized nanofibers. TEM image from core–shell nanofibers demonstrated the core-sheath structure of nanofibers prepared by coaxial electrospinning.

### 3.2 Release studies *in vitro* and *in vivo*

**Fig. 3:**
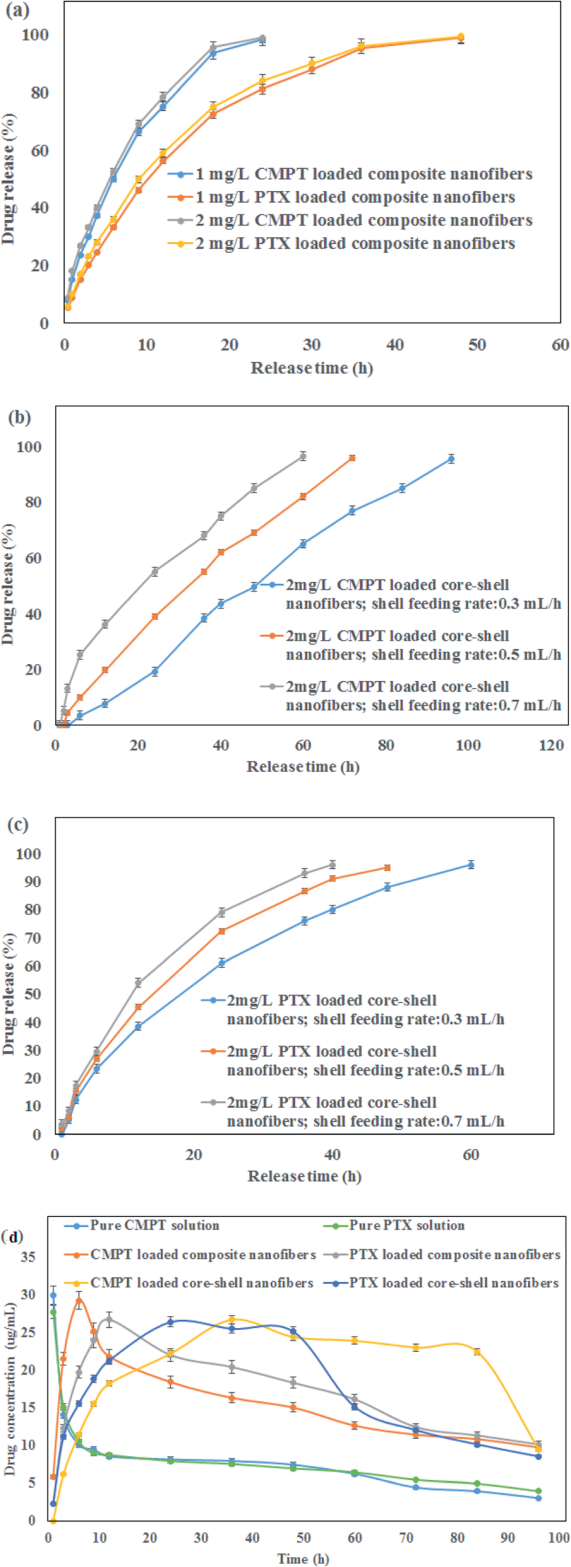
*In vitro* release profiles of (a) PTX and CMPT from PVA/κ-carrageenan/CMPT/Au/pegylated PU/PTX composite nanofibers, (b) CMPT from core–shell nanofibers, (c) PTX from core–shell nanofibers and (d) *in vivo* release profiles of PTX and CMPT from composite and core–shell nanofibers during 96 h.

DEE (%) and DLE (%) are evaluated for PTX and CMPT-loaded nanofibers which results are presented in Table 1(a). The higher DEE than 95% for both PTX and CMPT loaded core–shell nanofibers confirmed an effective loading of anticancer drugs into the core–shell nanofibers. The lower DEE and DLE for drug-loaded composite nanofibers compared to drugs-loaded core–shell nanofibers could be attributed to the removing unattached molecule on the nanofiber surface. As expected, DLE was increased by increasing the initial drug content. There were no significant differences between the DEE of 1 mg L^−1^ and 2 mg L^−1^ anticancer drugs-incorporated nanofibers. The release behavior of PTX and CMPT from PVA/κ-carrageenan/CMPT/Au/pegylated PU/PTX composite and core–shell nanofibers produced by various shell feeding rates is implemented under physiological pH (pH: 7.4) (Fig. 3a–c). The obtained results suggested that prepared composite fibers exhibited the drug sustained-release profiles for PTX and CMPT in terms of initial burst release, followed by a sustained release period for composite nanofibers and a sustained release without initial burst release from core–shell nanofibers. There was no significant difference between the release behavior of 1 and 2 mg L^−1^ PTX and CMPT from composite nanofibers. Therefore, the loading of 2 mg L^−1^ PTX and CMPT did not change the release pattern but different rates compared with 1 mg L^−1^ PTX and CMPT-loaded nanofibers, and it is possible to control the release rate of PTX and CMPT from nanofibers by adjusting the concentration of drugs. On the other hand, the time durations for PTX & CMPT release from core–shell nanofibers with 0.3, 0.5 and 0.7 mL h^−1^ shell feeding rate were 60, 48, 40 h & 96, 72, 60 h, respectively. Because of higher drug loading efficiency and more sustained release F4 formulation with core–shell structure showed the best result and was selected for *in vivo* release studies. The release data of PTX from composite and core–shell nanofibers best described using Peppas' equation (Table 1(b)). The values of release exponent (*n* = 0.35, 0.32 and 0.29 for CMPT) were smaller than 0.45 from core–shell nanofibers which indicated that CMPT molecules were fled from the core–shell nanofibers following the Fickian mechanism. The linear release achieved for CMPT release from core–shell nanofibers demonstrated an excellent fitting of release data with the zero-order pharmacokinetic model. The “*n*” values for PTX release from both composite and core–shell nanofibers and “*n*” values higher than 0.45 for CMPT release from composite nanofibers confirmed the non-Fickian diffusion mechanism.

(a) Drug loading efficiency of synthesized composite and core–shell nanofibers and (b) pharmacokinetic parameters of 2 mg L^−1^ CMPT and PTX release from composite and core–shell nanofibers (*n* = 5)

**Table:** 

(a)				
Sample	Drug	Concentration (mg L^−1^)	DEE (%)	DC (%)
Composite (F_1_)	CMPT	1	92.5 ± 0.5	2.628 ± 0.0142
	PTX	1	93.4 ± 0.7	2.653 ± 0.0199
Composite (F_2_)	CMPT	2	93.1 ± 0.8	5.290 ± 0.0455
	PTX	2	94.8 ± 0.7	5.386 ± 0.0397
Core–shell (F_3_)	CMPT	1	98.2 ± 0.3	2.790 ± 0.0085
	PTX	1	97.9 ± 0.4	2.781 ± 0.0114
Core–shell (F_4_)	CMPT	2	98.8 ± 0.2	5.614 ± 0.0114

**Table:** 

(b)								
Nanofibrous carrier	Drug	Zero-order		Higuchi		Korsmeyer–Peppas		
		*K*_0_ (h^−1^)	*R* ^2^	*K*_H_ (h^−0.5^)	*R* ^2^	*n*	*K*_KP_	*R* ^2^
Composite nanofibers	CMPT	0.5682	0.949	6.125	0.962	0.798	4.564	0.991
	PTX	0.4651	0.932	4.589	0.958	0.685	4.251	0.994
Core–shell nanofibers	CMPT	0.1842	0.989	2.897	0.941	0.293	2.251	0.990
Shell feeding rate: 0.3 mL h^−1^	PTX	0.4180	0.948	4.052	0.960	0.625	4.155	0.991
Core–shell nanofibers	CMPT	0.1925	0.990	3.134	0.928	0.324	2.351	0.992
Shell feeding rate: 0.5 mL h^−1^	PTX	0.4428	0.946	4.389	0.953	0.666	4.655	0.991
Core–shell nanofibers	CMPT	0.1989	0.992	3.379	0.944	0.352	2.846	0.993
Shell feeding rate: 0.7 mL h^−1^	PTX	0.4859	0.938	4.827	0.955	0.702	4.989	0.991

The *in vivo* PTX and CMPT (10 mg of each drug) release profiles from nanofibers are presented in Fig. 3d. 5 mL of 2 mg mL^−1^ PTX and CMPT solutions were used as control. Using pure PTX and CMPT solutions, plasma concentrations of PTX and CMPT came to their highest within 1 h and lower to average level at about 6 h for both anticancer drugs. When rats were fed with composite nanofibers, the blood concentration of CMPT and PTX reached the highest values of 29.3 ± 0.05 μg mL^−1^ and 26.8 ± 0.03 μg mL^−1^ in 6 h and 12 h and reduced slowly within 24 h and 48 h, respectively. Additionally, owing to the drug concentration gradient distribution from the inner layer to shell layer and then granted by core–shell nanofibers, which stores most drug in the inner layer, the curve of CMPT and PTX release from core–shell nanofibers exhibited a sustained release for about 84 h and 48 h, respectively due to the continuous release of drugs from core–shell nanofibers.

### 3.3 Cell viability

**Fig. 4:**
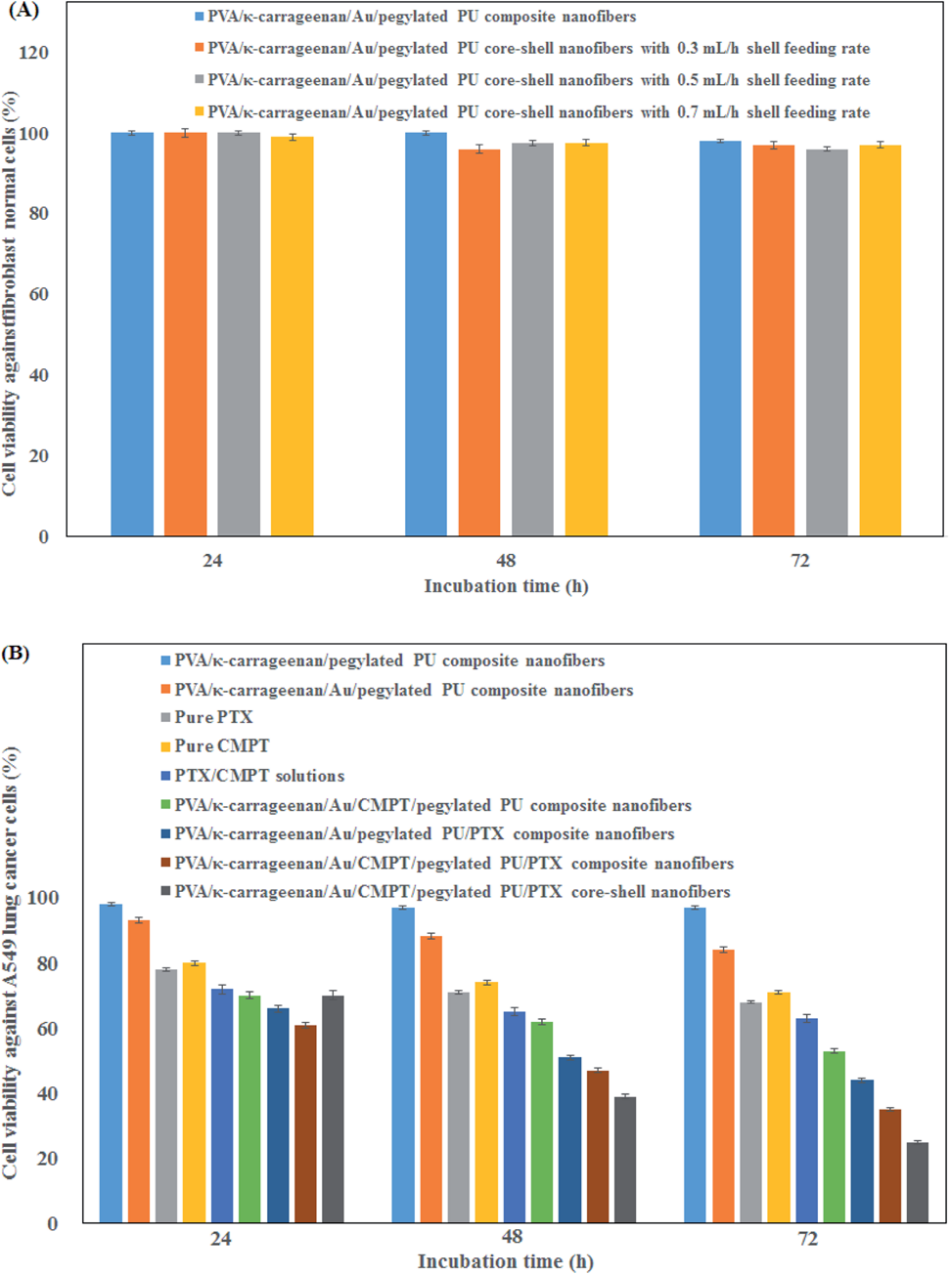
Cell viability of synthesized composite and core–shell nanofibers toward (A) L929 normal cells and (B) A549 lung cancer cells.

The cell viability of synthesized composite and core–shell nanofibers toward L929 normal cells is implemented to investigate the biocompatibility of synthesized nanofibers. The results are illustrated in Fig. 4A. The cell viability was higher than 90% for both composite and core–shell nanofibers, confirming the high biocompatibility of the synthesized nanofibrous samples. The cytotoxicity of PTX and CMPT loaded-nanofibers against A549 lung cancer cell lines is presented in Fig. 4B. The cancer cells were alive in the presence pure nanofibers without Au nanoparticles and anticancer drugs. A gradual decrease in the cell viability was observed after loading Au nanoparticles into the nanofibers, which indicated the anticancer activity of Au nanoparticles against A549 lung cancer cells. Whereas, the cell viability was reduced to 53%, 44% and 35% after treating A549 cancer cell lines with nanofibers containing CMPT, PTX and CMPT/PTX. The maximum cytotoxicity was about 75% in the presence PVA/κ-carrageenan/CMPT/Au/pegylated PU/PTX core–shell nanofibers (both core and shell feeding rates: 0.3 mL h^−1^). The sustained release of CMPT and PTX from nanofibers resulted in more cytotoxicity of core–shell nanofibers than composite nanofibers. *In vitro* cytotoxicity results showed that its inhibition effect on tumor cells by the PVA/κ-carrageenan/CMPT/Au/pegylated PU/PTX core–shell nanofibers was remarkable, but the toxicity for normal cells was slight. Similar trends are reported by other researchers.^49,50^ The simultaneous incorporation of Au nanoparticles and co-delivery of PTX and CMPT into the core–shell nanofibers led to effective treatment of A549 lung cancer cells.

### 3.4 *In vivo* studies

**Fig. 5:**
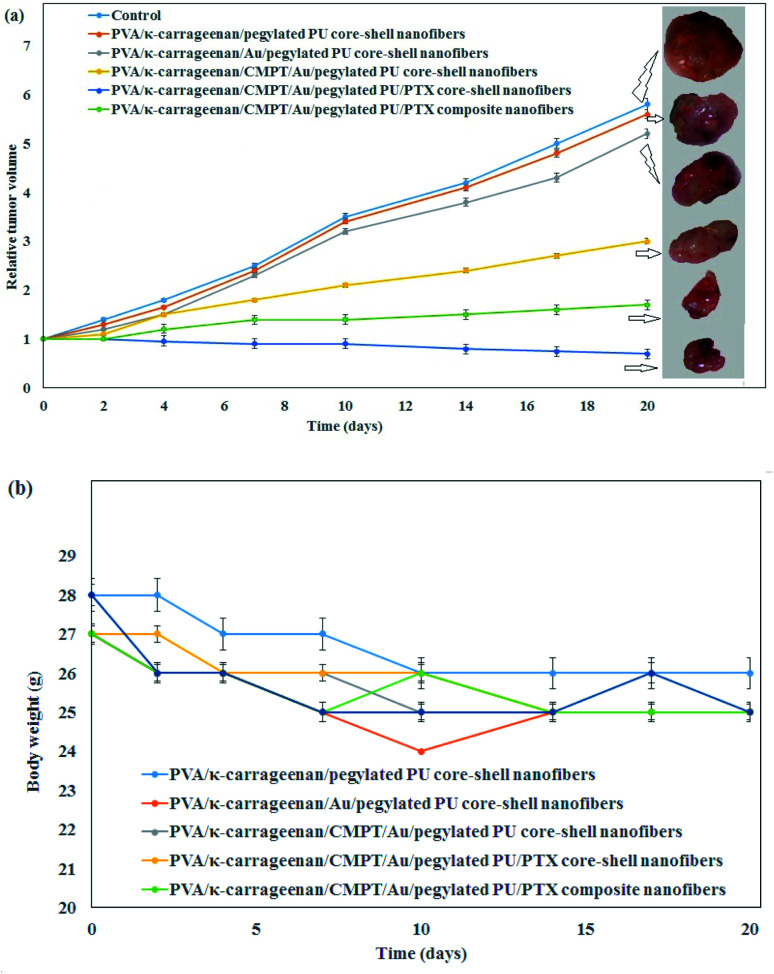
(a) *In vivo* antitumor efficacy of the PVA/κ-carrageenan/CMPT/Au/pegylated PU/PTX composite, and core–shell nanofibers and (b) mice body weight changes (number of trials = 3).

The *in vivo* antitumor efficacy of the PVA/κ-carrageenan/CMPT/Au/pegylated PU/PTX composite and core–shell nanofibers was investigated on the A549 tumor-bearing mice with an initial tumor volume of 100 mm^3^ which the results of tumor volume change and the weight of mice after treating with nanofibers are presented in Fig. 5. The pure nanofibers were selected as control. As shown in Fig. 5a, the maximum tumor inhibition was occurred in the presence PVA/κ-carrageenan/CMPT/Au/pegylated PU/PTX core–shell nanofibers compared to pure core–shell nanofibers, Au-loaded core–shell nanofibers, and composite nanofibers. The results indicated the synergistic effects of Au nanoparticles and anticancer drugs on tumor inhibition. Among the above four groups, the pure core–shell nanofibers did not show any tumor inhibition rate after 20 days. Furthermore, the nanofibers containing PTX and CMPT presented more effective tumor inhibition than Au nanoparticles loaded-nanofibers which revealed that the continuous release of CMPT and PTX from nanofibers could exert a better effect on the reduction of tumor volume and enhancement of tumor inhibition. Therefore, PVA/κ-carrageenan/CMPT/Au/pegylated PU/PTX core–shell nanofibers could be an effective localized delivery system for lung cancer treatment. The investigation of mice^'^s body weight after therapy revealed that no significant change occurred for all mice after injecting tumors for 20 days (Fig. 5b). For comparison of the capability of the synthesized core–shell nanofibers for the treatment of lung cancer cells with other nanofibrous scaffolds, Qiu *et al.*^51^ prepared the mesoporous silica nanoparticles/doxorubicin hydrochloride (DOX)-loaded poly(l-lactic acid) nanofibers (PLLA/DOX@MSNs) against human spca-1 lung cancer cells. The maximum cell viability was 73.10%. The release trend of DOX from PLLA/DOX@MSNs composite nanofiber was similar to the release profiles of CMPT and PTX from PVA/κ-carrageenan/CMPT/Au/pegylated PU/PTX composite nanofibers (initial burst release followed by a long period of slow-release). The inhibition rate of titanocene dichloride-loaded PLLA nanofibers was 68.2% (240 mg L^−1^ drug concentration).^52^ The maximum cell viability of A549 lung cancer cells by the chitosan/PLLA/TiO_2_/DOX/GO nanofiber and a magnetic field was about 82% after 86 h.^53^ Therefore, the co-incorporation of Au nanoparticles and anticancer drugs into the nanofibers demonstrated an optimal therapy effect on the tumor inhibition without changing the body weight.

## 4. Conclusion

In this work, Au nanoparticles, CMPT and PTX anticancer drugs were successfully incorporated into the PVA/κ-carrageenan/pegylated PU composite and core–shell nanofibers. The applied voltage of 20 kV, tip–collector distance of 12 cm and feeding rate of 0.3 mL h^−1^ were optimum values for the fabrication of PVA/κ-carrageenan/CMPT/Au/pegylated PU/PTX composite nanofibers with an average diameter of 225 nm. The mean fiber diameter for core–shell nanofibers produced under shell feeding rates of 0.3, 0.5, and 0.7 mL h^−1^ were 330, 520 and 640 nm, respectively. The higher DEE than 95% for PTX and CMPT confirmed an effective loading of anticancer drugs into the nanofibers. Time durations for PTX vb. CMPT release from core–shell nanofibers with 0.3, 0.5 and 0.7 mL h^−1^ shell feeding rates were 60, 48, 40 h *vs.* 96, 72, 60 h, respectively. The linear release is achieved for CMPT release from core–shell nanofibers. The PTX release data of composite, core–shell nanofibers and CMP release data of composite nanofibers were best described using the Peppas' equation. *In vivo* release studies indicated that rats fed with core–shell nanofibers, the blood concentration of CMPT and PTX reached the highest values of 26.8 ± 0.04 μg mL^−1^ and 26.5 ± 0.05 μg mL^−1^ in 36 h and 24 h and kept in the constant values between 36–84 h and 24–48 and finally reduced after 84 h and 48 h, respectively. The L929 cell viability higher than 90% for both composite and core–shell nanofibers confirmed the high biocompatibility of the synthesized composite and core–shell nanofibrous samples. The maximum cytotoxicity was 75% in the presence PVA/κ-carrageenan/CMPT/Au/pegylated PU/PTX core–shell nanofibers. *In vivo* antitumor efficacy results of A549 tumor-bearing mice treated with composite and core–shell nanofibers demonstrated the best effect on the reduction of tumor volume and enhancement of tumor inhibition without changing the mice's weight in the presence PVA/κ-carrageenan/CMPT/Au/pegylated PU/PTX core–shell nanofibers.

## Abbreviations

ANOVA: Analysis of variance
BDO: 1,4-Butanediol
CMPT: Camptothecin
DOX: Doxorubicin hydrochloride
DEE: Drug entrapment efficiency
DLE: Drug loading efficiency
FTIR: Fourier transform infrared
Au NPs: Gold nanoparticles
GO: Graphene oxide
HDI: Hexamethylene diisocyanate
HPLC: High-performance liquid chromatography
MOFs: Metal–organic frameworks
DMF: *N*,*N*-Dimethylacetamide
PTX: Paclitaxel
PBS: Phosphate buffered saline
PEG: Poly(ethylene glycol)
PLA: Poly(lactic acid)
PLGA: Poly(lactic-*co*-glycolic acid)
PU: Polyurethane
PVA: Polyvinyl alcohol
SEM: Scanning electron microscope
TEM: Transmission electron microscope
XRD: X-ray diffraction

## Conflicts of interest

There are no conflicts to declare.

## Supplementary Material
